# Comparing intensive care treated primary, secondary and tertiary sepsis with respect to length of stay, organ failure and mortality: a pilot study

**DOI:** 10.1186/cc12413

**Published:** 2013-03-19

**Authors:** S Castegren, M Jonasson, J Sjölin, M Castegren

**Affiliations:** 1 Centre for Clinical Research Sörmland, Uppsala University, Uppsala, Sweden; 2 Medical Sciences, Uppsala University, Uppsala, Sweden

## Introduction

It may be hypothesized that a patient's inflammatory status might affect the clinical picture and outcome of a subsequent septic insult. To test this hypothesis, patients were categorized according to previous inflammatory activity and analyzed with respect to clinical picture and outcome in a retrospective study.

## Methods

The Swedish ICU registry was used to identify all adult patients diagnosed with severe sepsis or septic shock in a single Swedish ICU during 2006 to 2011. The charts were reviewed and patients categorized into four groups based on whether the patient had suffered systemic inflammatory-inducing insults previous to the septic episode. The patients were categorized as: (A) primary sepsis, no recent (<30 days) trauma/infection, ongoing systemic inflammatory or immunosuppressive disease or malnutrition; (B) secondary sepsis, recent (<7 days) trauma/infection or systemic inflammatory disease, but no immunosuppressive disease or malnutrition; (C) tertiary sepsis, >1 fulfilled criteria of secondary sepsis during the last 10 days or immunosuppressive disease or malnutrition; and (D) patients not attributable to A to C. After the categorization, physiological and laboratory parameters during the ICU stay as well as therapy and outcome were extracted from the charts. Only significant differences (*P *0.05) are presented; Mann-Whitney and chi-squared tests were used.

## Results

A total of 273 charts were reviewed. In total, 242 were categorized into A (*n *= 126), B (*n *= 79) or C (*n *= 37). D (*n *= 31) consisted mainly of patients with hematological malignancies (*n *= 12) and patients with chemotherapy or immunosuppressive treatment (*n *= 14). The groups differed in length of stay with A< B< C. During the first 3 days the SOFA score was higher in A compared with C and in B compared with C. The duration of antibiotic therapy was longer in both B and C compared with A. There were no differences in 28-day mortality (A: 34/126 = 27%, B: 30/79 = 38%, C: 15/37 = 40%); however, the proportions of patients dying between days 8 and 28 were higher in B (14/63 = 22%) and C (7/29 = 24%) compared with A (5/95 = 5%).

## Conclusion

In this retrospective material it was possible to categorize 88.6% of all patients as having primary, secondary or tertiary sepsis. The categories differed in clinical picture at presentation as well as in outcome. A prospective study is warranted to validate the results of this study.

